# Computational version of the correlation light-field camera

**DOI:** 10.1038/s41598-022-25780-4

**Published:** 2022-12-10

**Authors:** Thomas Gregory, Matthew P. Edgar, Graham M. Gibson, Paul-Antoine Moreau

**Affiliations:** 1 School of Physics and Astronomy, University of Glasgow, Glasgow, G12 8QQ UK; 2 Department of Physics, National Cheng Kung University, Tainan, 70101 Taiwan; 3 Center for Quantum Frontiers of Research and Technology, NCKU, Tainan, 70101 Taiwan

**Keywords:** Imaging and sensing, Optical sensors

## Abstract

Light-field cameras allow the acquisition of both the spatial and angular components of the light-field. The conventional way to perform such acquisitions leads to a strong spatio-angular resolution limitation but correlation-enabled plenoptic cameras have been introduced recently that relax this constraint. Here we use a computational version of this concept to acquire realistic light-fields images using a commercial DSLR Camera lens as an imaging system. By placing the image sensor in the focal plane of a lens, within the camera we ensure the acquisition of pure angular components together with the spatial information. We perform an acquisition presenting a high spatio-angular rays resolution obtained through a trade off of the temporal resolution. The acquisition reported is photo-realistic and the acquisition of diffraction limited features is observed with the setup. Finally, we demonstrate the refocusing abilities of the camera.

## Introduction

Since the discovery of quantum ghost imaging^1^ and the realisation that it can be reproduced qualitatively both through the use of classical correlations^2^ and through computational means^3,4^ a number of classical and computational correlation imaging techniques have emerged that exhibit advantages in terms of implementation when compared to conventional imaging^5,6^. Among these techniques is the single-pixel camera^4^, where filtering patterns are displayed on a programmable spatial modulator, and the resulting correlation signal is detected by a single pixel photo-sensor. The emergence of this technique led to the realisation that an image can indeed be acquired with a single pixel detector, and can be used in a wide range of applications^7^. This can present advantages when no spatially resolved detectors are accessible (or are prohibitively expensive), for example when imaging at exotic wavelengths^5,7^, or at very precise timing resolution^8,9^. But if a single pixel detector is in this context sufficient to acquire conventional images a natural question to ask then is whether similar techniques could also present advantages in conditions where spatially resolved detectors are both accessible and affordable? In other words can the use of multiple-pixels together with such correlation imaging techniques lead to an advantage in terms of the extracted information?

In various experimental examples the use of a few photo-diodes has been shown to present such an advantage. A first example uses four spatially separated photo-diodes to obtain four images of a scene, seen from the exact same point of view but under different illumination conditions leading to a difference in the shading of the scene. This allowed 3D reconstructions to be performed using a shape from shading algorithm^10^. A second example uses three photo-diodes to perform a coloured image reconstruction from the individual red green and blue signals^11^, this was later modified to add an infrared detector and perform simultaneous real-time visible and infrared video acquisitions^12^. On the other hand, the use of a camera together with spatial correlations have been achieved to perform ghost acquisitions of a temporal signal. This was done computationally^13^, with pseudo thermal correlations^14^, and also with quantum correlations^15^.

But crucially, the combined use of spatial correlations imaging techniques and of the detection by a camera allows to access supplemental spatial quantities of the light. D’Angelo et al.^16,17^ have introduced the concept of correlation plenoptic cameras by suggesting that schemes with two cameras could harness pseudo-thermal correlations to perform light-field imaging^18,19^. In their scheme one camera is used to enable the correlation based imaging while the second camera ensures the acquisition of angular information of the light-field. The same group was able to implement this proposal experimentally^20^, demonstrating the possibility of obtaining diffraction limited acquisitions through this technique. These concepts were also extended to the use of quantum correlations^21^. Additionally, the phase image of an object is another example of supplementary imaging information that can be accessed through the combined use of spatial correlations enabled imaging techniques and of a spatially multiplexed detection in a Fourier domain^22^. However, the main limitation of the aforementioned techniques, lies in use of physical correlations, which leads to complex imaging schemes and a limited image quality. Indeed, such physical correlations are not perfectly controllable and therefore are sub-optimal to perform an image reconstruction^23–25^. Additionally, physical correlation techniques rely on the detection of a correlated reference beam of light, which leads to supplementary experimental noise. In contrast, computational techniques based on pattern projections offer great potential for low noise imaging^3,4^, not only does it allow the use of a controllable, and potentially optimal, pattern projection, but it also removes the need to detect a noisy reference beam.

On the other hand, coded light-field imaging techniques have been developed that are based on the use of a coded aperture^26–29^ with limited angular resolution and overall number of light rays acquired. Other techniques such as capturing a focal stack can also in principle be a way to access a light-field reconstruction^30,31^. However, such techniques require a primary depth map estimation based on ’empirical’ treatment of the images, or prior depth assumptions such as quasi continuity about the scene^32^. This is because some spatio-angular information is lost when moving from light-field data to refocused data.

High spatio-angular resolution light-field acquisition with a realistic scene remains to be demonstrated with a camera that is self-standing i.e. that do not require the control of the illumination. We report here such an acquisition by implementing the concept of correlation plenoptic imaging in its a computational version with a setup allowing to access the exact angular domain by using a conventional CCD and a conventional DSLR camera lens as an imaging system. By such a spatially resolved detector together with a programmable spatial light modulator (SLM), which in our implementation is a Digital Micro-mirror Device (DMD), we acquire photo-realistic images of a 3-dimensional scene and report the observation of diffraction limited features at certain refocusing depths. We demonstrate that the technique can lead to very high dimensional light-field acquisitions by performing single acquisitions of $256\times 256$ images (angular resolution) composed of $128\times 128$ pixels (spatial resolution). The technique generally makes use of a camera with $N_\alpha$ pixels and a spatial light modulator with $N_x$ pixels to lead to a light-field reconstruction with $N_\alpha N_x$ rays. This is obtained through a spatio-temporal resolution trade off identical to the one involved in conventional single-pixel camera acquisitions: we need to acquire multiple frames over time for various projected patterns. Nevertheless, the increased spatio-angular resolution represents a quadratic advantage in contrast to the conventional light-field acquisitions^18,19^ whose total spatial and angular resolution is limited by the number of pixels of the camera in use, and which requires a trade-off between the spatial and angular resolution. There are ways to partially circumvent that exact limit through employing other trade-offs, but such techniques are still limited and do not lead to a major increase in the total resolution^33^.

We note that for both the conventional light-field technique using a microlens array and in the presently reported technique a spatio-angular resolution and SNR trade off will exist as an increase of the spatio-angular resolution will lead to a decrease of the SNR at a given incoming optical power. This is because in both cases the electric signal recorded will become noisier as the intensity per measurement decreases. In regimes where the detector noise dominates the trade-off will be similar in both techniques, however in regimes dominated by the illumination noise, the single pixel technique is known to scale more poorly with the resolution^34,35^. It is therefore expected that for the technique presented here the scaling of the SNR with the angular resolution will be similar to the one in the conventional micro-lens array technique, but also that the SNR should degrade with the spatial resolution faster than in micro-lens technique in illumination noise dominated regimes. This is because the spatial resolution is obtained here through single pixel reconstruction. However, it has been shown that such a drawback can be mitigated for a given acquisition time through using micro-scanning techniques^34^. Finally, it is very important to note that a fair comparison of SNRs between conventional and single pixel acquisition techniques would essentially require technical arguments, as the sensors used in both cases would fundamentally be different and exhibit noises of different nature. For example, to date no giga-pixel cameras are commercially available. Such a camera would be necessary to reproduce the acquisition reported here through a micro-lens array technique. As a result it would not be possible to make a fair prediction as to the noise exhibited by such hypothetical cameras and the SNR of the corresponding light-field acquisition.

Finally, we show the quasi-continuous and realistic refocusing abilities of our system that are enabled by the high angular resolution.

## Results

**Figure 1:**
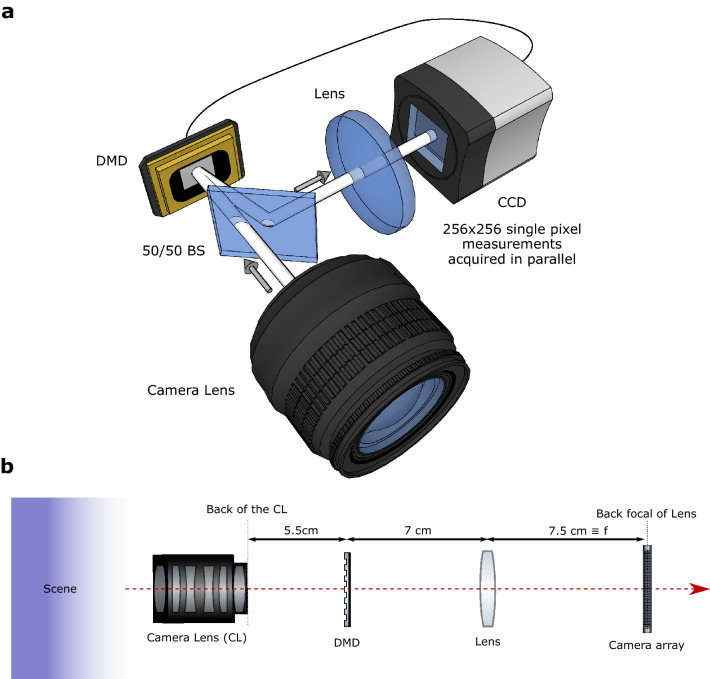
Computational light-field Imaging setup. A Camera lens is used to image a scene onto a Digital Micro-mirror Device (DMD), and that can perform a single pixel measurement on each pixels. The light reflected off the DMD pattern is then reflected by a 50/50 beamsplitter (BS) and sent onto a CCD camera set to image the spatial frequencies in the scene. By displaying Hadamard patterns on the DMD, and acquiring camera images for each displayed pattern, one can perform a single-pixel-like light-field. (**a**) 3D representation of the light-field camera with 50/50 BS allowing the use a DMD around normal incidence (**b**) unfolded optical configuration with the relative position of the various optical components along the optical axis. The DMD is here represented in transmission for simplicity and the BS is not represented.

As shown in the setup presented in Fig. 1, the proposed technique consists of using a programmable DMD placed at the back-focal plane of a large aperture camera lens. The DMD operates as a binary device, rapidly actuating each micromirror to one of two states. We can display 2D binary patterns on the DMD and the light reflected along one path off of the DMD is collected in an imaging arm by using a 50/50 beamsplitter. The imaging arm is composed of a simple lens and a CCD camera placed in its focal plane. The CCD camera is thus collecting exact angular information about the light-field, and the DMD on the other hand can be used to filter spatial information about the scene. Note that the position of the second lens and CCD camera system relative to the DMD is unimportant as their role is simply to produce an optical Fourier transform. The crucial point here is to ensure that the camera is placed in the back focal plane of the second lens in such a way that the camera will truly acquire pure angular information making the light-field acquisition optimal. The camera sensor is indeed that way placed in the far-field (acquiring spatial frequencies). In that configuration, the camera pixels are performing pure angular ray-information acquisition while the DMD pixels perform image position ray-information acquisition. This allows no information overlap between the two measurements performed in two Fourier conjugated planes, thus maximising the relaxation of the spatio-angular resolution constraint.

In Fig. 1b we further detail the position of the important optical components along the optical axis. It can be seen that the camera is in the back-focal plane of the lens. The camera is thus in a Fourier plane compared to the DMD, the DMD pixels will correspond to image pixels while the camera pixels acquire pure angular components.

The calibration of the optical system is performed as follows. We first ensure that the DMD is in a plane of focus of interest, to do that we display a pattern on the DMD, and turn the focusing ring of the camera lens so that the plane of interest in the scene is in focus in the plane where the DMD patterns appear in focus as well. We then ensure that the lens is in the back focal plane by acquiring far away images with the camera (optical infinity), moving the lens in front of the camera until the focus is obtained. We then place the lens-camera system in the setup, moving the ensemble close enough to the DMD such that no vignetting is observed on the camera. This ensures, that the last lens is not cutting some of the spatial frequencies, and that the whole camera plane can be used to measure these angles.

The acquisition is performed by displaying a full set of Hadamard patterns on the DMD and synchronously acquiring images with the camera. Although it may not always be generally optimal to reduce the acquisition noise^36^ the Hadamard pattern set is the set of choice to perform image reconstruction due to their easy mathematical construction and the fact that they form an orthogonal basis^37,38^. Using an orthogonal basis avoids redundancy in the acquisition, so that each measurement provides new information about the scene. The patterns are furthermore democratised to avoid prospecting too widely varying signal intensity values, and to stay within the dynamic range of the camera^25^.

For each of the camera pixels one can extract a temporal signal and perform a conventional single pixel image reconstruction^7^. We do this in the following way, let $A_{i,k}$ be a sequence of $N_\alpha$ orthonormal pattern pairs, where pixels can have values of $\pm 1$ (*i* is the pixel number and *k* is the pattern number), the corresponding differential signal obtained on pixel *j* of the CCD camera is $S_{k,j}$. The reconstructed image of the scene for a particular CCD pixel *j* (determining the Point of View of the reconstruction) is noted $O_{i,j}$ and is simply estimated as

1 $$\begin{aligned} O_{i,j}=\frac{1}{N_\alpha }\sum _{k=1}^{N_\alpha }{S_{k,j}A_{i,k}} \end{aligned}$$

**Figure 2:**
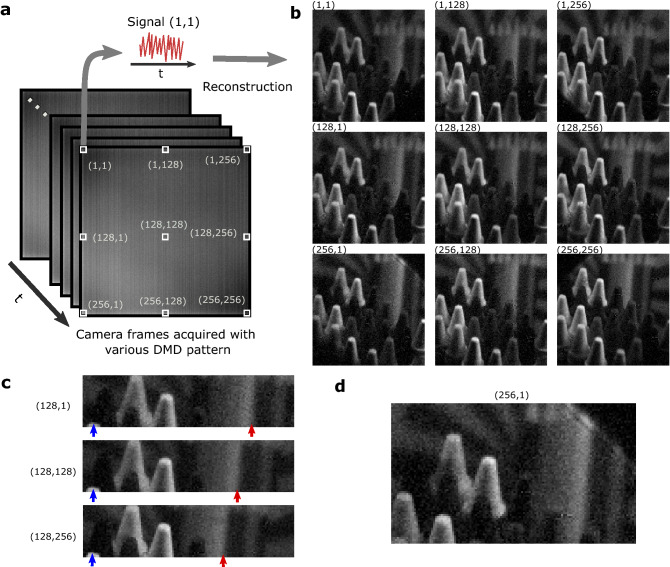
A computational light-field acquisition. (**a**) Schematic principle of the reconstruction. The set of camera frames acquired with different patterns are used to extract signals. A single signal is obtained by selecting in each of the images a particular $4\times 4$-binned pixel. With each of the $256\times 256$ obtained signals one can reconstruct an image of the scene as seen from a particular POV through a single pixel camera reconstruction. (**b**) Nine of the $256\times 256$ reconstructed images obtained for a single light-field acquisition. These images correspond to a reconstruction of the same scene from different points of view, obtained by using camera binned-pixels with coordinate (*x*, *y*). (**c**) Detail extracted from three reconstructed images. The red and blue arrows highlight the position of two features in the different images. (**d**) Detail showing a vignetting effect observable on the images reconstructed using the four pixels in the corner of the CCD camera.

Because the light captured by a particular pixel of the camera corresponds to a particular angle of propagation in the DMD plane, the scene will ultimately appear as being seen from this particular angle in the reconstructed image. This is much like in conventional light-field imaging^18,19^ in which an array of micro-lenses determine the spatial resolution and where the direction of propagation of the light is detected by a subgroup of pixels leading to the reconstruction of a particular point of view. The difference in our implementation here being that each camera pixel is now able to perform this angular measurement for each of the DMD pixels (i.e. for each of the image pixels), that is because through the use of the single-pixel acquisition and reconstruction technique we can untangle the contribution of each of the image pixels within a single CCD camera pixel signal. As a consequence using only two $n^2$ pixels devices, we obtain $n^2$ different light-field images composed each of $n^2$ pixels, that is a total of $n^4$ effective light-field rays. This is in contrast with conventional light-field acquisitions for which the use of an $n^2$ pixel camera will lead only to a $n^2$ total effective light-field pixel reconstruction. Note, however, that the present method requires the acquisition of multiple images and cannot be performed as a ’snapshot acquisition’, the number of frames to acquire can be reduced through compressive sensing techniques. A schematic of this reconstruction process is shown in Fig. 2a.

In practice we extract $1024\times 1024$ pixel images from the CCD camera and bin the pixels by groups of $4\times 4$ in order to improve the SNR by statistically reducing the relatively high camera noise, for this purpose we also acquire 16 images per Hadamard pattern ($128\times 128$ pixels) so as to wash out the effects of the camera noise. By doing so we reconstruct a full light-field acquisition composed of $256\times 256$ points of view of the scene with images composed of $128\times 128$ pixels, that is an effective total acquisition of one Giga light-field rays. Note that we limit the DMD to $128\times 128$ pixels i.e. below its native resolution of $1024\times 768$ to reduce the total acquisition time. Increasing the image resolution is in principle possible but would require to display an increased number of patterns thus increasing the acquisition time. In Fig. 2b we show nine out of the $256\times 256$ images acquired in a single acquisition. A navigation within these $256\times 256$ different views can also be seen in Visualization 1. Figure 2c illustrates the change of point of view obtained in the resulting images. The three images reconstructed from different binned CCD camera pixels are juxtaposed and the red and blue arrows highlight two particular features in the scene. One can see that depending on the depth of the object compared to the plane of optical focus, the positions of the features are shifted from one image to the other, illustrating the change of angular point of view under which the scene is seen in the different images. Finally in Fig. 2d we show the presence of a vignetting effect in the images reconstructed with the CCD camera pixels situated in the corner of the sensor.

We want to formalise here the tradeoff that our technique requires in terms of acquisition time. Consider a lens array light-field acquisition using a camera with $N_{pix}$ pixels, if all the pixels were optimally used, one could obtain an acquisition with $N_{pix}=N_xN_\alpha$ rays, each of the $N_x$ image pixels (corresponding to the numbers of micro-lenses) would be recorded for each of the $N_\alpha$ angular point of views, in a single shot with acquisition time $\Delta t$. To obtain the same acquisition with our method, one would need to use $N_\alpha$ camera pixels and project $N_x$ different patterns. That is an acquisition time of $N_x\Delta t$ that we would obtain through raster scanning or Hadamard pattern projection. This acquisition time can however be considerably reduced by using compressed sampling^4^. However, our technique allows to break the constraint that the acquisition should be limited to $N_{pix}$ rays. We can now acquire up to $N_\alpha =N_{pix}$ angular components for each of the $N_x$ image pixels, $N_x$ being limited only by the number of DMD pixels and by the $N_x\Delta t$ acquisition time it requires. Concerning the noise performance in such scenarios, as discussed in the introduction, in regimes dominated by the detector noise, the noise performance of our single pixel light-field scheme acquired in a time $N_x\Delta t$ will be similar to that of a lenslet array scheme leading to the acquisition of the same number of rays in $\Delta t$. They will both scale similarly with an increased number of acquired rays $N_xN_\alpha$. To formalise this, consider a light-field inputting on a camera with total illumination power *P*. A camera with exposure time $\Delta t$ would record a mean signal of $S=P\Delta t/N_{pix}$ on a single pixel, where $N_{pix}$ is the number of pixels. In the same way, in lenslet array light-field acquisitions the signal is spread over the $N_{pix}=N_xN_\alpha$ pixels the mean signal will therefeore be $S=P\Delta t/(N_xN_\alpha )$. The average signal to noise ratio in such an acquisition will be given by

2 $$\begin{aligned} SNR_{la}=\frac{P\Delta t}{N_xN_\alpha \sigma _c} \end{aligned}$$

Where $\sigma _c$ is the noise per pixels of the camera. Now in the single pixel light-field realisation, we consider for simplicity that raster scanning is applied, at each of the $N_x$ pattern projection steps a single pixel is lit. The signal oncoming onto the camera is therefore divided by $N_x$. Then that signal is spread onto the $N_\alpha$ pixels recorded by the camera. We will therefore have a similar scaling of the signal $S=P\Delta t/(N_xN_\alpha )$ and consequently of the SNR:

3 $$\begin{aligned} SNR_{sp}=\frac{P\Delta t}{N_xN_\alpha \sigma _c} \end{aligned}$$

In either case increasing the number of acquired rays will degrade similarly the SNR. And would necessitate the increase of the scene illumination power *P* by the same factor to retrieve a similar SNR or an increased acquisition time. Of course it should be noted that given the fact that the acquisition time is already increased by the spatio-temporal trade-off in the case of the single pixel acquisition, may render it difficult to further increase the acquisition time, and it may be desirable to sacrifice some of the SNR instead. This is an advantage for the lenslet scenario whose constraints on acquisition time are lesser. Though we should again note that the achieved ray resolution reported here $N_xN_\alpha =1$ Giga is simply not currently achievable in a lenslet array scenario. Moreover, it is likely that the noise $\sigma _c$ of an hypothetical 1 Giga pixel camera would be widely greater than that of a $N_\alpha =256\times 256$ pixel camera (the effective camera resolution used in our acquisition).

One of the advantages of our technique is that it allows a great depth of field to be accessed, which allows numerical refocusing along a large range of depths. The depth of field (*DOF*) gives an estimation on the range of positions along the optical axis in the object space that will appear in focus in an image. In the case of the present setup this depth of field is increased through angular post-selection that is performed when reconstructing an image using the signal extracted from a given pixel on the camera. The camera pixel post selection acts as a numerical-aperture-limiting pupil placed upstream of the image sensor in a conventional image acquisition. It is expected that the small size of the pixels leads to a large *DOF* of the reconstructed images, thereby allowing some details to be brought into focus which could not be obtained with a conventional image acquisition or with a conventional light-field acquisition. Indeed for a conventional light-field acquisition the number of camera pixels per microlens limits the number of accessible points of view, each of these few pixels will then occupy a relatively large area in terms of the different angular components it collects. The *DOF* can be expressed in the following way^39^:

4 $$\begin{aligned} DOF =\frac{2NL_o^2f^2B}{f^4-N^2L_o^2B^2} \end{aligned}$$

Where $L_o$ is the object position relative to the camera lens entrance pupil, f is the focal length of the lens placed in front of the CCD camera, *B* is the acceptable blur diameter criterion and *N* is the f-number. One can observe the role of the f-number in Eq. (4) that shows that the depth of field is increased when the optical system f-number is increased.

The f-number is an image-space, infinite-conjugate quantity^39^, that can be linked to the image space numerical aperture $NA _i$ in the following way, under the small angle approximation:

5 $$\begin{aligned} N\approx \frac{1}{2 NA _i} \end{aligned}$$

In our implementation, $NA _i$ will effectively be limited by the single pixel angular post selection. Let $s_p$ be the size of the effective camera pixels used for the reconstruction, and $f_c$ be the focal length of the camera lens. In the image space, the pixel will act as the angular acceptance pupil and the associated numerical acceptance will be, under the small angle approximation:

6 $$\begin{aligned} NA _i\approx \frac{s_p}{2f_c} \end{aligned}$$

This leads to the following expression for the effective f-number for the ’single pixel’ reconstructed images:

7 $$\begin{aligned} N\approx \frac{f_c}{s_p} \end{aligned}$$

In the context of our experiment an f-number of up to $N\sim 5000$ can potentially be obtained by using unbinned CCD pixels. Through the reported acquisition, using $4\times 4$ pixel binning, we achieved an f-number of $N\sim 1300$ and when the full CCD sensor signal is integrated over $1024\times 1024$ camera pixels, the obtained effective f-number is $N\sim 5$.

Such a large f-number $N\sim 1300$ in our realisation allows for the detection of diffraction-limited features as we can show theoretically. Indeed, the diffraction limited spot expected with our setup in the plane of the DMD (image plane) is given by

8 $$\begin{aligned} d=\frac{\lambda }{2 NA }=\lambda N \sim 500\cdot 10^{-6}\times 1300 \sim 0.65\,{\text {mm}} \end{aligned}$$

This is significantly greater than the ’instrument response function’ feature size that in our case is determined by the size of the super-pixels used in the projected Hadamard patterns, that span $6\times 6$ physical DMD micro-mirrors. Indeed, the DMD used presents a pixel pitch of $13.7\upmu {\text {m}}$ giving an effective images pixel size of $s_i=6\times 0.0137=0.0822\,{\text {mm}} \ll d$. Our acquisition is therefore quantitatively predicted to be diffraction limited.

**Figure 3:**
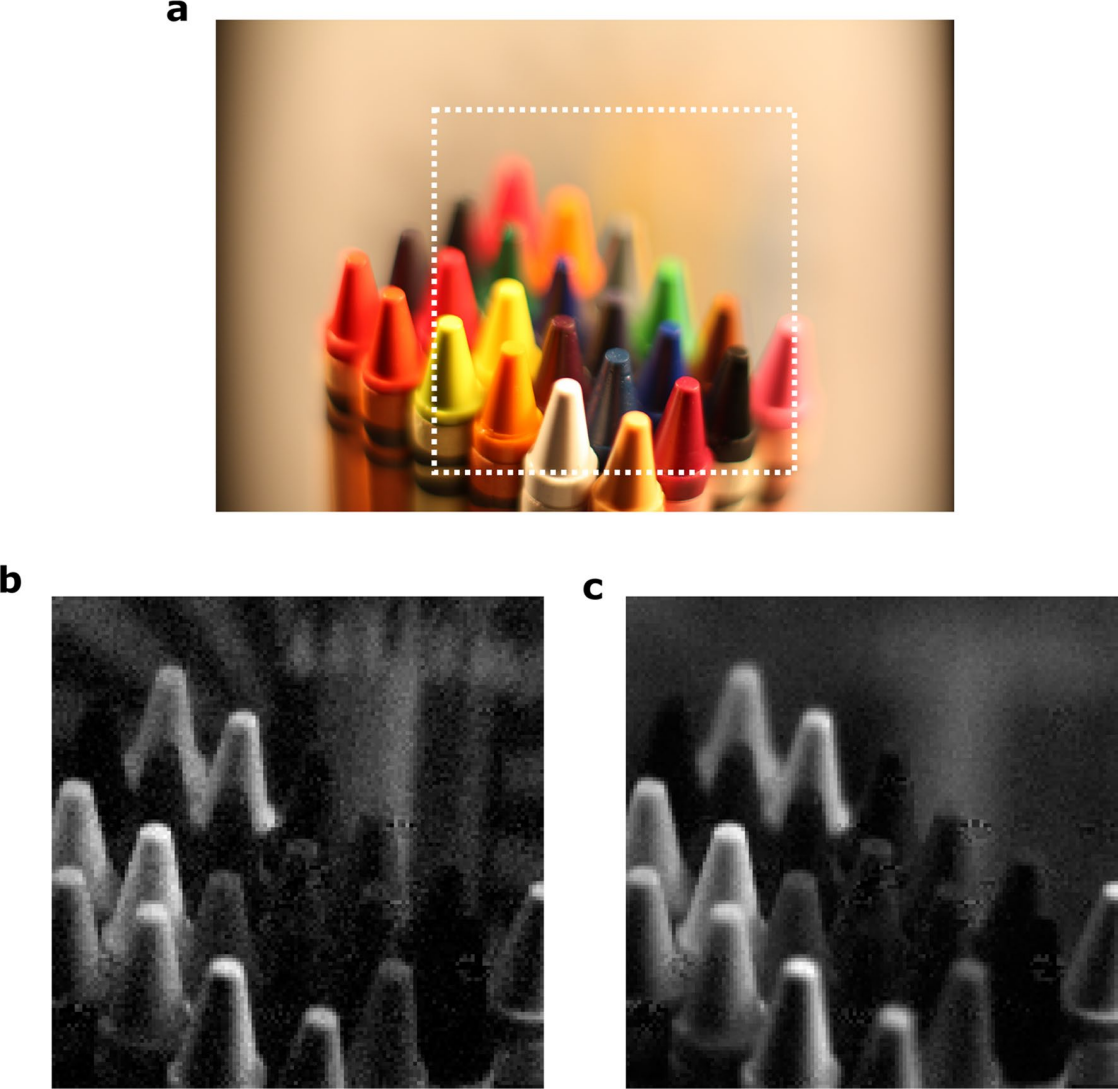
Comparison of the DOF in various images. (**a**) Photograph of the same scene obtained with a DSLR camera placed behind the camera lens placed in the same configuration as during the light-field acquisitions. The expected f-number in this context is $\sim 1.4$. The dashed line box highlights the field of view obtained in the light-field acquisitions. (**b**) Central light-field reconstruction (128,128) using our method. The expected f-number in this context is $\sim 1300$. (**c**) Integrated single-pixel reconstruction using the same light-field acquisition and the full-frame images intensity as signal to perform the reconstruction. The expected f-number in this context is $\sim 5$.

In Fig. 3 we show a comparison of three images of the same scene. Figure 3a is an image acquired with a DSLR camera, Fig. 3b is one of the $256\times 256$ images acquired with our system and Fig. 3c is an image corresponding to the same light-field acquisition, but for which the signal used to generate the reconstruction was the integrated intensity measured on the whole CCD camera sensor. All three acquisitions were performed using the same camera lens in the same configuration. As expected the depth of field seen on Fig. 3b, that is, the depth of field accessible through our light-field reconstruction is greater than the one accessible to a conventional image acquisition with the same camera lens. This exemplifies the increased DOF effect due to the pixel post selection described above. Figure 3c illustrates how the depth of field can actually be manipulated by choosing the size of the binned pixels.

**Figure 4:**
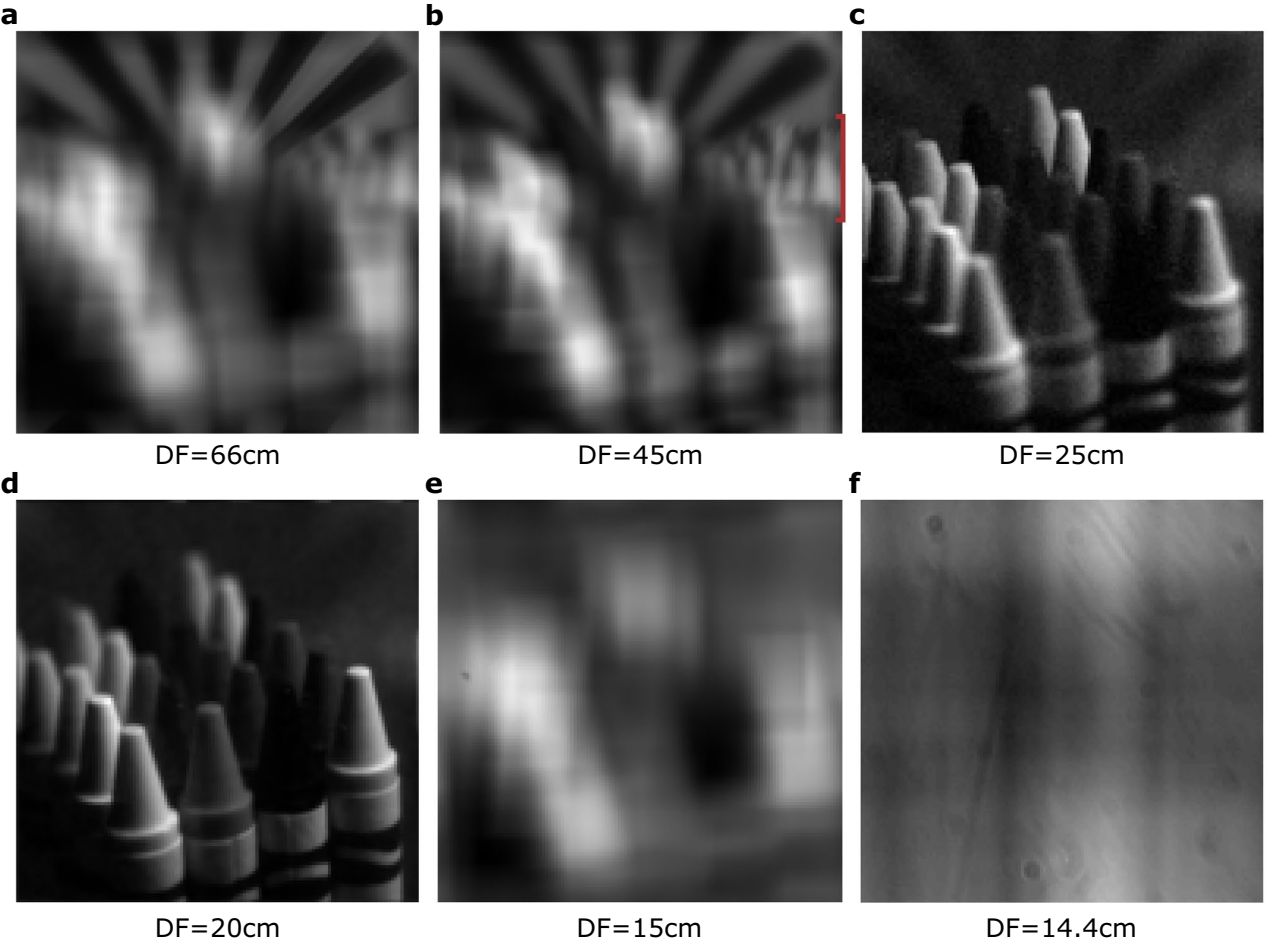
Refocusing at various depths. We use a second single computational light-field acquisition of $256\times 256$ POV images of $128\times 128$ pixels to perform a computational refocusing at various depths in the scene. The depth of focus (DF) is the distance between the camera objective and the depth at which we intend to focus in the object space. (**a**) Focusing on the spoke target background. (**b**) Focusing on the colour pencils (their position is highlighted by a red bracket on the right side of the picture). (**c**) Focusing on the farthest crayon. (**d**) Focusing on the nearest crayon. (**e**) Focusing in front of the scene. (**f**) Focusing on dust particles that are not part of the scene but on optical surfaces on the camera lens that are re-imaged on planes close to infinity in the image space.

We further demonstrate the refocusing capabilities of the light-field acquisitions we obtained. The refocusing is performed by treating the full one Gigapixel data, simply through a translation of each of the images in each of the two dimensions, by an amount proportional to the coordinates of the binned camera pixels used to reconstruct each image. The translated images are then summed altogether. One can set the value for the smallest translation i.e the difference in image translation from one binned camera pixel to the next. The different values will correspond to different depths of re-focus. The larger this parameter the farther from the optical focus the numerical focus will be. Examples of refocusing at different depths are presented in Fig. 4, a continuous refocusing is also shown in Visualization 2. In Fig. 4c one can observe the presence of noise that is not present in the other refocused images. The depth of focus used in this context is the actual optical focus plane of the camera lens, so that the different images are here simply summed without translation. Because there is a fixed pattern noise shared by all the images, this adds constructively in this image and is washed out in the others because the fixed pattern is then translated independently in the different images before they are summed. One can see this effect as well when observing stereoscopic images using two POV images extracted from our acquisitions. We present an anaglyph 3D in the supplementary text, and also give the two POV images used that can be displayed on a 3D screen or headset (see supplementary text). When observing this stereoscopic image one can see the depth observation 3D effect which shows that our images are candidates to perform 3D stereoscopic reconstructions. Moreover, the noise can even be located in depth approximately at the central depth of the crayons, this is where the camera lens was set to focus.

It can also be observed in Fig. 4f that when refocusing close to the object space focal plane, diffraction limited dust particles can be observed on the images, with a clear Airy disk appearance. Indeed, the great numerical apertures accessible, through computational light-field imaging, means that the factor preventing focusing closer and closer to the object focal plane will be the then greater and greater magnification of the objects leading to the observation of diffraction limited features, thus reaching a limit similar to the one reported in the physical correlation technique presented in ref.^20^. The observation of such diffraction features is an illustration that the depth of field accessible through the computational light-field imaging that we report allows imaging of optical planes where the resolution limit is no longer bounded by the sensor properties. This is demonstrated by the fact that the Airy disk pattern is apparent and not blurred by a de-focus effect, i.e. by signal integration over the binned pixel, and also that such diffraction spots are not present in images acquired with a conventional camera in the same condition Fig. 3a. We can analyse the size of these spots that are 9 super-pixels large in the image 4f, this corresponds to a size of $s_a=9\times s_i=9\times 0.0822\sim 0.74\,{\text {mm}}$ which is qualitatively comparable to the theoretical predicted diffraction-limited spot size $d=0.65\,{\text {mm}}$.

## Discussion

As shown above the computational light-field camera that we have developed allows the acquisition of photo-realistic images with a high spatio-angular resolution. By allowing higher angular resolutions to be accessed compared to a conventional light-field camera the principles of this technique can be applied to present a number of advantages. We list a few in the following.

First, the technique presents potential for super-resolution based on microscanning reconstruction methods^40^. Because an object at a certain depth moves from one point of view to the next relative to the pixel mesh, this can be harnessed to perform a microscanning reconstruction.

A second advantage of the technique lies in the great depth of field that it allows access to. This enables very realistic refocusing along a large range of depth. When the angular resolution of a light-field acquisition is not good enough, the out of focus background of a refocused image often presents an unrealistic periodic repetition of the background objects rather than a continuous fuzzy out of focus object. By removing the spatio-angular resolution trade-off, the computational light-field camera can resolve such issues.

A third advantage of our technique can be found in the continuity of the views compared to microlens light-field acquisitions makes the light-field acquisition in itself more realistic. This enables the possibility to realistically navigate within the light-field space as illustrated in Visualization 1.

Finally, in the context of 3D reconstruction, the use of a large depth of field and the associated high angular resolution may also generate great interest. Using the light-field data extracted from the present camera would lead to limited z resolution at large distances due to the fact that the two extreme points of view will be ultimately limited by the camera lens numerical aperture. Nevertheless, the technique presented here has a clear advantage at very short and mid-range distances: the multiplicity of the different points of view represents as many planes to perform a 3D reconstruction along various different depths. Take for example two adjacent angular pixels the change in the scene will not be significant enough to perform a 3D reconstruction on distant objects, but will be useful for very near objects. On the other hand using reconstructions from two CCD camera pixels further apart will be perfect for 3D reconstruction at mid-range. Using the full light-field acquisition one could therefore perform 3D reconstructions of a scene on a continuum of short and mid depth ranges, let alone the possibility to use an additional device to also reach long distance ranges.

On the other hand, one of the drawbacks of the technique is the speed of the acquisitions. In our demonstration, we were limited to acquire CCD images at a rate around 50 Hz due to the limited camera frame rate and the intensity fluctuations of the incandescent light in use that follows the electrical fluctuations of the power supply network. Moreover, the large noise of our CCD camera led us to acquire 16 images for each pattern displayed on the DMD, additionally for each pattern we were also displaying its negative, in order to perform a differential measurement and remove further light intensity fluctuations^41,42^. For these reasons the total acquisition time for a single one Gigapixel acquisition was of the order of around 3 h ($128\times 128\times 2\times 16=524{,}288$ images at 50 Hz). But it needs to be noted that the DMD in use could in theory be used at 20 kHz, similarly the camera in use here can reach $\sim$500 Hz once lighting fluctuations are solved, commercially available high speed cameras can in fact reach the DMD speed of 20 kHz in terms of frame rate at $>256\times 256$ pixels^43^. Finally, it has been shown that $\sim 700$ displayed patterns are sufficient to perform $128\times 128$ pixel image acquisitions^44^. We therefore expect that the acquisition time could in principle be greatly reduced if such improvements can be implemented while limiting the effect of the noise.

## Methods

In the experimental implementation we used a Canon EF50mm f/1.4 USM Camera lens. The single lens placed in front of the CCD camera was a single biconvex 2” diameter lens with a focal length of f=75mm. The CCD camera used for the acquisition of angular images was an Optronis CL600x2/M, with a resolution of $1280\times 1024$ presenting a pixel size of 14 $\upmu {\text {m}}$. Finally the DMD used was a ViALUX High Speed V Module (V-7000) with a DMD chip number DLP7000 presenting $1024\times 768$ pixels.

## Supplementary Information

Supplementary Information 1.

Supplementary Video 1.

Supplementary Video 2.

## Data Availability

All data needed to evaluate the conclusions in the paper are present in the paper and the Supplementary Materials.
