# Supplementary material for: Computational version of the correlation light-field camera
Source: Sci Rep. 2022 Dec 10;12:21409. doi: 10.1038/s41598-022-25780-4 (PMC9741593; doi:10.1038/s41598-022-25780-4)
Supplement: Supplementary file 1 — Supplementary Information 1. [file 41598_2022_25780_MOESM1_ESM.pdf]

Supplementary material: Computational version of the Correlation Light-Field Camera

Thomas Gregory[1], Matthew P. Edgar[1],
Graham M. Gibson[1], Paul-Antoine Moreau[2,3,*]

[1]School of Physics and Astronomy, University of Glasgow, G12 8QQ, UK
[2]Department of Physics, National Cheng Kung University, Tainan 70101, Taiwan
[3]Center for Quantum Frontiers of Research and Technology, NCKU, Tainan 70101, Taiwan

*Corresponding author:
Email : pa.moreau@gs.ncku.edu.tw

We describe here the supplementary materials associated with our realisation of a computational light-field camera. We first quickly describe the two supplementary videos and in addition provide stereoscopic images obtained by selecting two different images among the set of images obtained within a single light-field acquisition.

1 Supplementary video S1: Single Light Field Acquisition.

In this video we show a navigation within a one Gigapixel acquisition with 256×256 different 128×128 pixels images. The video is the acquisition of a graphical user interface software we developed. We highlight the presence of diffraction limited dust particles at the end of the video. The objects in the scene are moved around when changing the point of view. The movement is relative to the camera lens optical plane of focus, that is the only depth plane not moving in the scene.

2 Supplementary video S2: Refocusing.

In this video we show the quasi continuous refocusing at various depth of the scene captured in a Gigapixel light-field acquisition. The video is the acquisition of a graphical user interface software we developed. We highlight the observation of diffraction limited dust particles when refocusing close to the object focal plane of the camera lens.

3 3D stereoscopic viewing

We present here two images selected within the set of points of view obtained through a computational light-field acquisition. These images can be used to display a stereoscopic image. In Fig. S1 we give the left eye image and in Fig. S2 we give the right eye image. These images can be extracted and displayed on the 3D stereoscopic screen of a headset. Furthermore, we present a red-cyan anaglyph 3D in Fig. S3 constructed with these two images. When observing this stereoscopic image one can see the depth observation 3D effect which shows that our images are candidates to perform 3D stereoscopic reconstructions. Finally when observed on a screen or with an headset one can actually observe the localisation of the noise in depth. The noise is indeed situated around the central depth of the crayons, within the camera lens optical plane of focus.

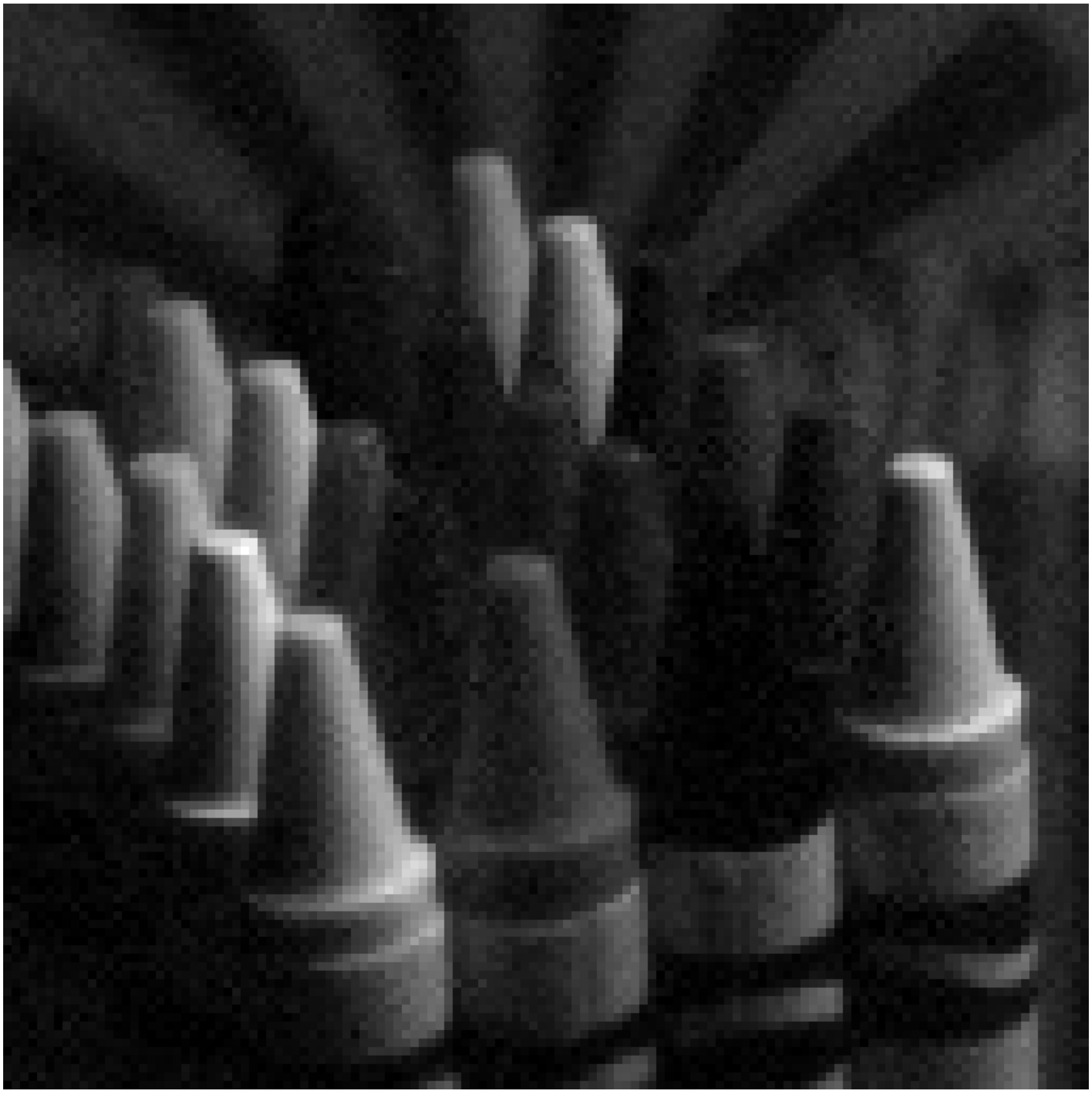

Figure S1: **Left eye image.**

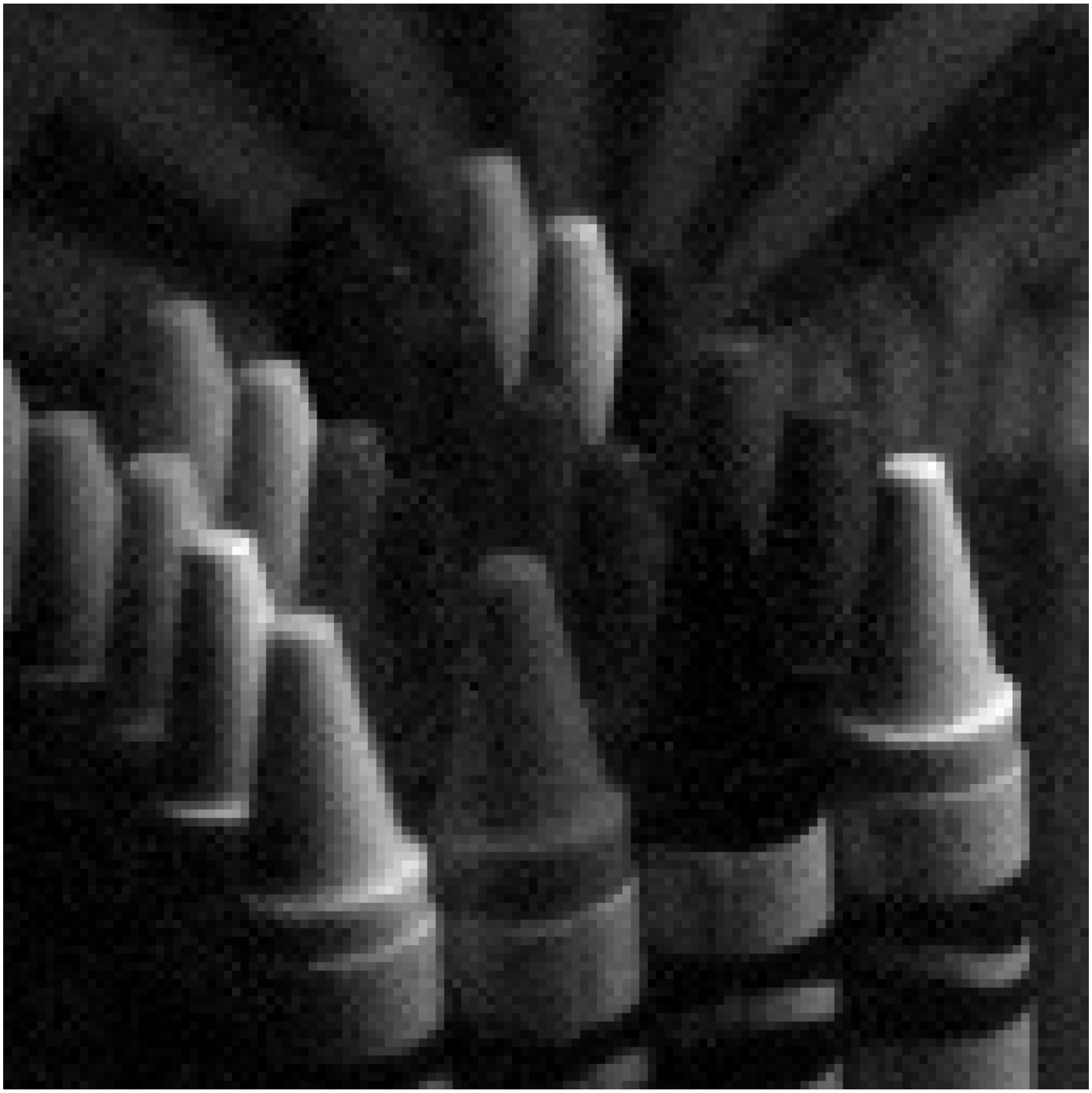

Figure S2: **Right eye image.**

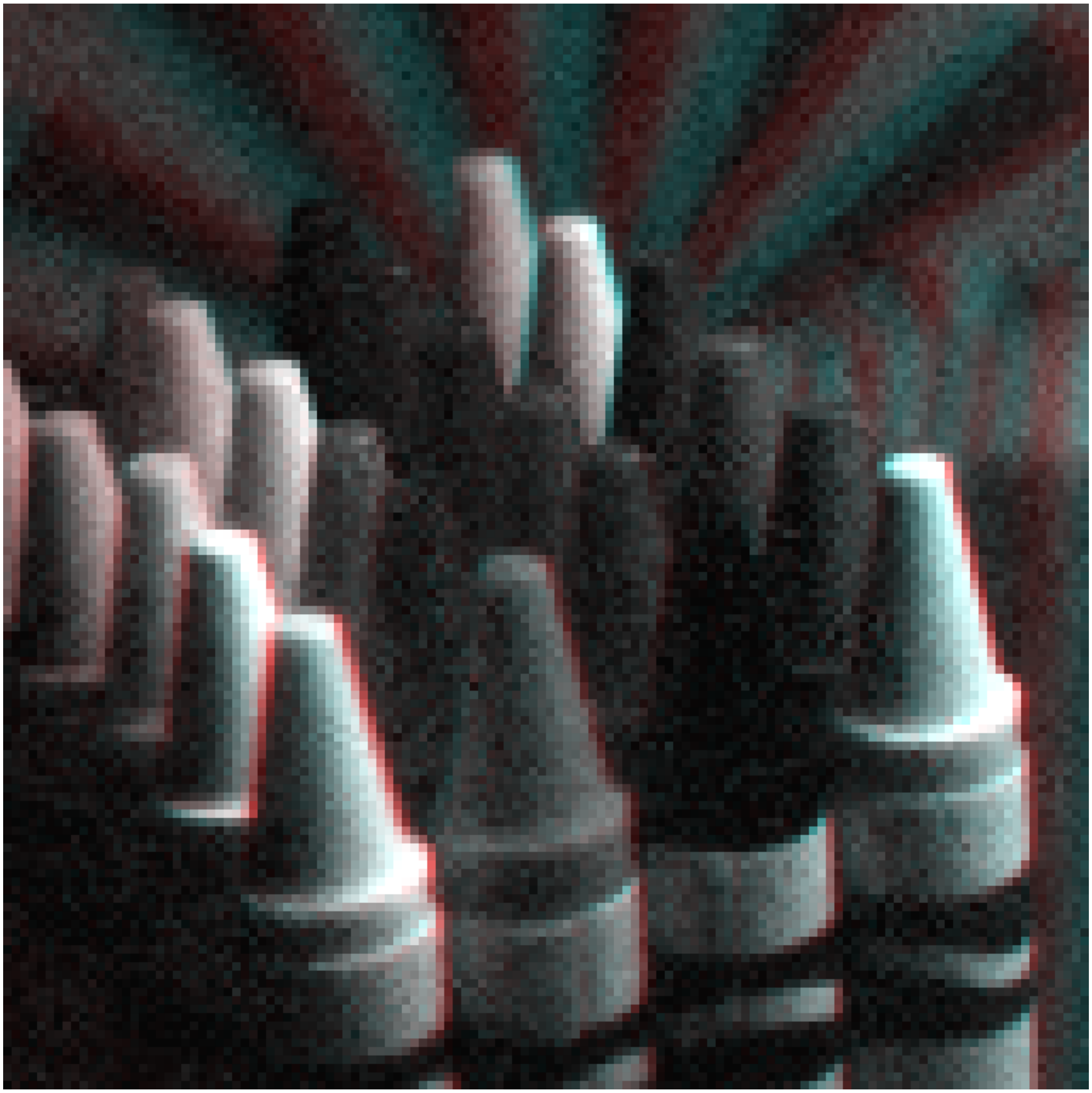

Figure S3: **Red-cyan anaglyph 3D.** Constructed with two images extracted from a computational light-field acquisition.
